# Thymosin *β*4 Prevents Oxidative Stress, Inflammation, and Fibrosis in Ethanol- and LPS-Induced Liver Injury in Mice

**DOI:** 10.1155/2018/9630175

**Published:** 2018-07-11

**Authors:** Ruchi Shah, Karina Reyes-Gordillo, Ying Cheng, Ravi Varatharajalu, Joseph Ibrahim, M. Raj Lakshman

**Affiliations:** ^1^Lipid Research Laboratory, VA Medical Center, 50 Irving Street NW, Washington, DC, USA; ^2^Department of Biochemistry and Molecular Medicine, The George Washington University Medical Center, 2300 I Street NW, Washington, DC, USA; ^3^Institute of Biomedical Sciences, The George Washington University, 2300 I Street NW, Washington, DC, USA

## Abstract

Thymosin beta 4 (T*β*4), an actin-sequestering protein, is involved in tissue development and regeneration. It prevents inflammation and fibrosis in several tissues. We investigated the role of T*β*4 in chronic ethanol- and acute lipopolysaccharide- (LPS-) induced mouse liver injury. C57BL/6 mice were fed 5% ethanol in liquid diet for 4 weeks plus binge ethanol (5 g/kg, gavage) with or without LPS (2 mg/kg, intraperitoneal) for 6 hours. T*β*4 (1 mg/kg, intraperitoneal) was administered for 1 week. We demonstrated that T*β*4 prevented ethanol- and LPS-mediated increase in liver injury markers as well as changes in liver pathology. It also prevented ethanol- and LPS-mediated increase in oxidative stress by decreasing ROS and lipid peroxidation and increasing the antioxidants, reduced glutathione and manganese-dependent superoxide dismutase. It also prevented the activation of nuclear factor kappa B by blocking the phosphorylation of the inhibitory protein, I*κ*B, thereby prevented proinflammatory cytokine production. Moreover, T*β*4 prevented fibrogenesis by suppressing the epigenetic repressor, methyl-CpG-binding protein 2, that coordinately reversed the expression of peroxisome proliferator-activated receptor-*γ* and downregulated fibrogenic genes, platelet-derived growth factor-*β* receptor, *α*-smooth muscle actin, collagen 1, and fibronectin, resulting in reduced fibrosis. Our data suggest that T*β*4 has antioxidant, anti-inflammatory, and antifibrotic potential during alcoholic liver injury.

## 1. Introduction

Chronic alcohol consumption is associated with the development of alcoholic liver disease (ALD), which is one of the leading causes of morbidity and mortality worldwide [1]. The progression of ALD comprises a spectrum of features including hepatic steatosis, inflammation, fibrosis, and cirrhosis that may eventually lead to death [2]. Although, excessive alcohol consumption is the major cause of end-stage liver disease, epidemiological studies have shown that alcohol alone is not enough for the pathogenic transformation of alcoholic hepatosteatosis to the more severe forms of ALD. There is increasing evidence that endotoxin (lipopolysaccharide (LPS)) found in the gut is an important contributor to the onset of liver damage associated with ALD [3–6]. Studies have shown that LPS is commonly elevated in the blood of alcoholics [7] and in certain animal models exposed to alcohol [8]. Thus, it is now well accepted that alcoholic liver injury involves ethanol- (EtOH-) induced oxidative stress and acetaldehyde as the first hit and EtOH-mediated endotoxin release due to leaky gut as the second hit, resulting in inflammation and subsequently fibrosis.

Liver fibrosis is a wound healing response to chronic liver damage that is marked by the activation of hepatic stellate cells, the main fibrogenic cells of the liver. Activated HSC are proliferative, proinflammatory, and fibrogenic with induced ability to synthesize and deposit large amounts of ECM proteins ([9]) accompanied by overexpression of genes that confer the myofibroblastic phenotype such as platelet-derived growth factor receptor beta (PDGF-*β*R), collagen 1, fibronectin, and de novo synthesis of alpha smooth muscle actin (*α*SMA) ([9, 10]), resulting in the formation of scar tissue.

Thymosin beta 4 (T*β*4) is a 43-amino acid polypeptide initially isolated from calf thymus [11]. It belongs to the family of 15 members with a highly conserved amino acid sequence [11]. It is an immune-modulating molecule that has drawn significant attention in regenerative medicine [12]. T*β*4 prevents inflammation and fibrosis and promotes wound healing in the eye, skin, and heart [13–17]. In the eye, it promotes corneal reepithelization after injury [13]. It also inhibits the inflammation after alkali injury with sodium hydroxide [18]. Overall, it prevents inflammation by blocking the secretion of inflammatory cytokines and suppressing the activation of NF*κ*B [19]. In the heart, it prevents the formation of scar tissue after a myocardial infarction by enhancing the survival of myocardial tissue and endothelial cells, thus sustaining cardiac function and preventing scar formation [12, 14, 20]. T*β*4 also inhibits the appearance of myofibroblasts in a model system of wound healing [21]. It has also been reported that depletion of T*β*4 results in the activation and proliferation of HSC. We have previously shown that T*β*4 inhibited PDGF-*β* receptor expression and Akt phosphorylation resulting in an inhibition of HSC proliferation and migration [22]. Additionally, we also showed that T*β*4 protected against carbon tetrachloride-induced acute liver injury in rat [23]. Therefore, in this study, we investigate the effect of T*β*4 in alcohol-mediated liver injury and fibrosis.

## 2. Materials and Methods

### 2.1. Animals and Diet

Eight-week-old, wild-type female (females are more susceptible to alcohol-induced liver damage than males [24–26]) C57BL/6 mice (~25 g body weight) from Charles River, Wilmington, MA, were housed in pairs per cage in plastic cages, in a temperature-controlled room at 25°C with 12-hour light-dark cycle. All animals were fed a pelleted commercial diet (Purina Rodent Chow, #500, TMI Nutrition, St. Louis, MO, USA) during the first week of acclimation period after arrival. Experiments were performed according to the approved institutional animal care and use committee protocol. Mice were randomly divided into 6 groups of 4 rats each and were pair-fed Lieber-DeCarli control or EtOH liquid diets (36% total fat calories) with high-*ω*3 fatty acid (14.1% of calories as *ω*3 fatty acids) fish oil for 4 weeks. The diets are isocaloric, and their formulations are according to the modified method of Lieber and DeCarli [27] with the recommended normal nutrients, vitamins, and minerals according to AIN-93 diet [28]. Thus, 36% of the total energy of EtOH diet is from fat, 20% from protein, 36% from EtOH, and the rest from the carbohydrate. The corresponding isocaloric control diet has isoenergetic amounts of dextrin-maltose in place of EtOH. EtOH concentration in the liquid diet was gradually increased starting at 1% level on day 1 and reaching the 5% level over a 7-day period to allow the animals to adapt to EtOH in the diet. The mice fed with EtOH diets were also administered with a single dose of 5 g/kg body weight of EtOH orally by gavage, with or without LPS (2 mg/kg body weight, i.p.), 6 h prior to euthanizing the animal. This 2-hit model of EtOH- and LPS-induced liver injury was adapted from the 2-hit EtOH/LPS model by Hoek [4] and EtOH/binge model by Bertola et al. [29]. The acuteness on chronic EtOH/binge model follows the drinking pattern of many alcoholic hepatitis patients who have a background of chronic drinking as well as a history of recent excessive alcohol consumption (binge) [30]. T*β*4 was administered intraperitoneally as a daily dose of 1 mg/kg of body weight for 1 week prior to euthanizing the animal.

### 2.2. Plasma Liver Injury Markers

Plasma alanine aminotransferase (ALT) and aspartate aminotransferase (AST) were measured by using commercial kits (Teco Diagnostics, Anaheim, CA, USA).

### 2.3. Hematoxylin and Eosin Staining

Liver tissues were fixed and processed for staining with hematoxylin and eosin using routine protocol as previously described [31].

### 2.4. DCFDA ROS Assay

ROS levels were determined according to the manufacturer's instructions of a commercial kit that utilizes a fluorometric assay (Cell Bio Labs, San Diego, CA, USA). The cell-permeable fluorogenic probe 2′,7′-dichlorodihydrofluorescin diacetate (DCFH-DA) is diffused into cells and is deacetylated by cellular esterases to nonfluorescent 2′,7′-dichlorodihydrofluorescin (DCFH), which is rapidly oxidized to highly fluorescent 2′,7′-dichlorodihydrofluorescein (DCF) by ROS. The fluorescence intensity is proportional to the ROS levels within the cell.

### 2.5. Reduced Glutathione (GSH) Assay

0.1 g of tissue was weighed and washed with ice-cold saline and homogenized in 10 ml of ice-cold buffer (10 mM Tris-HCl, pH 7.2; 250 mM sucrose; 1 mM EDTA) containing 2× protease inhibitor cocktail. Reduced GSH was quantified according to the manufacturer's instructions (Sigma, St. Louis, MO, USA).

### 2.6. Total, Nuclear, and Cytosolic Protein Extraction

Total protein was extracted from liver tissue by homogenization in lysis buffer containing 1 mol/l Tris (pH 8), 5 mol/l NaCl, 0.5 mol/l EDTA, 0.5 mol/l NaF, 100 mmol/l sodium pyrophosphate, 100 mmol/l Na_3_VO_4_, and 200 mmol/l phenylmethylsulfonyl fluoride. Nuclear and cytosolic fractions were extracted using a commercial kit (Thermo Scientific, Rockford, IL, USA).

### 2.7. SDS-PAGE and Western Blot Analysis

Protein concentrations were determined by bicinchoninic acid assay (Pierce Chemical Rockford, IL, USA), and the indicated proteins were determined by Western blotting as previously described [32]. Mn-SOD, NF*κ*B, I*κ*B, PDGF-*β* receptor, and PPAR*γ* antibodies were purchased from Cell Signaling, Danvers, MA, USA; p-I*κ*B, *α*SMA, fibronectin, and lamin B1 antibodies were obtained from Abcam, Cambridge, MA, USA; and collagen1*α*2 and *β*-actin antibodies were purchased from Santa Cruz Biotechnologies, Santa Cruz, CA, and Sigma, St. Louis, MO, USA, respectively.

### 2.8. RNA Extraction and Quantitative RT-PCR

RNA from liver tissue was extracted using TRIzol reagent (Life Technologies, Carlsbad, CA, USA). cDNA templates were synthesized, and quantitative RT-PCR was carried out as previously described [32]. 40S ribosomal protein S14 was used as the standard housekeeping gene. Ratios of target gene and S14 gene expression levels were calculated by subtracting the threshold cycle number (*C*_t_) of the target gene from the *C*_t_ of 40S ribosomal protein S14 and raising 2 to the power of the negative of this difference. Target gene expression is expressed relative to 40S ribosomal protein S14 gene expression.

### 2.9. Sirius Red Staining

4–6 *μ*m thick sections were deparaffinized and rehydrated using xylene, 100% ethanol, 95% ethanol, and 70% ethanol for 3 min each and stained using Picro-Sirius Red staining kit (Abcam, Cambridge, MA). Picro-Sirius Red solution was applied to completely cover the tissue section and incubated for 1 hour and then rinsed twice in acetic acid solution and absolute alcohol. Sections were then dehydrated by dipping twice in absolute alcohol and then mounted with Eukitt quick hardening mounting medium (Sigma-Aldrich, St. Louis, MO). Picro-Sirius Red stains collagen 1 and collagen 3 fibers in the tissue section. Staining of collagen fibers was assessed based on five 20x magnification fields per animal per group in a blinded fashion using Zeiss 510 microscope (Carl Zeiss, Thornwood, NY). Percent of fibrosis was calculated based on the intensity of Sirius Red staining using ImageJ (NIH, Bethesda, MD).

### 2.10. Hydroxyproline Assay

Hydroxyproline content in liver tissue was measured colorimetrically using a commercial kit (Sigma, St. Louis, MO, USA).

### 2.11. Statistical Analysis

All experiments were performed in triplicate, and data are expressed as mean ± SE. Statistical differences between experimental groups were analyzed by Student's *t*-test, and *p* < 0.05 was considered to be significantly different (Microsoft Excel 2011, Microsoft, Redmond, WA, USA).

## 3. Results

### 3.1. T*β*4 Prevents EtOH- and LPS-Induced Liver Injury

**Figure 1:**
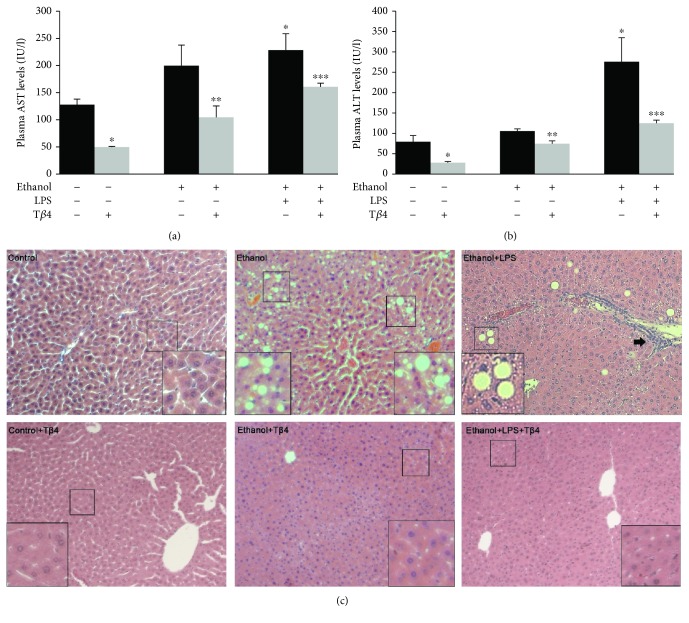
Effect of T*β*4 on EtOH- and LPS-induced liver injury. Biochemical analysis of plasma (a) AST, and (b) ALT, and (c) H&E staining of liver sections from various groups. Lipid droplets for steatosis and inflammatory infiltration in ethanol and ethanol + LPS groups are indicated in the insets and by arrows. Magnification, 20x. All values are means of triplicate experiments ± SE. ^∗^*p* < 0.05 versus control; ^∗∗^*p* < 0.05 versus EtOH; ^∗∗∗^*p* < 0.05 versus EtOH + LPS.

To investigate the extent of liver injury mediated by EtOH and LPS administration, we measured the liver injury markers, aspartate aminotransferase (AST) and alanine aminotransferase (ALT) in the various groups. AST and ALT are cytosolic enzymes of the hepatocyte and an increase in the levels of these enzymes in the blood reflects plasma membrane permeability, indicating cell death and tissue damage [33]. As illustrated in Figure 1(a), plasma AST was markedly increased by 56% in EtOH-treated group and 78% (*p* < 0.05) in EtOH + LPS group as compared to the control. Treatment with T*β*4 significantly lowered the level of AST by 46% (*p* < 0.05) and 30% (*p* < 0.05) in EtOH-treated mice in the absence or presence of LPS, respectively. Similarly, the plasma ALT level was nominally increased by 33% in the EtOH group but was significantly increased by 248% (*p* < 0.05) in EtOH + LPS group. These EtOH- and LPS-mediated increases were reduced by T*β*4 by 20% (*p* < 0.05) and 56% (*p* < 0.05) as compared to EtOH and EtOH + LPS groups, respectively. Moreover, histological analysis showed extensive hepatocellular damage induced by EtOH and LPS (Figure 1(c)). This is evidenced by the presence of lipid droplets indicating steatosis in the EtOH group, as well as necrosis and inflammatory infiltration in the EtOH + LPS group. These histopathological changes were ameliorated by T*β*4 treatment (Figure 1(c)).

### 3.2. T*β*4 Prevents Ethanol- and LPS-Induced Oxidative Stress

**Figure 2:**
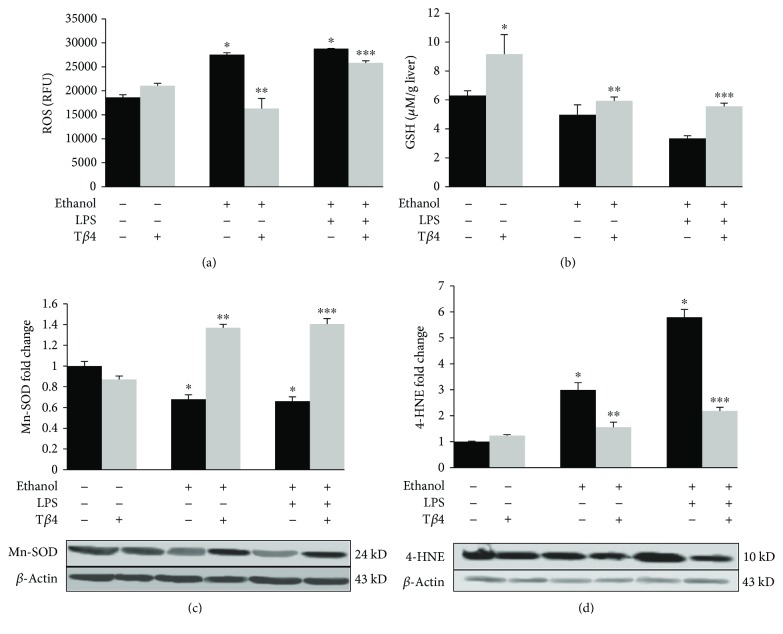
Effect of T*β*4 on EtOH- and LPS-induced hepatic oxidative stress. Liver tissue from various groups was used to determine (a) ROS, (b) reduced GSH, and total protein expression of (c) Mn-SOD and (d) 4-HNE. All values are means of triplicate experiments ± SE and were corrected for loading differences after reprobing with *β*-actin. ^∗^*p* < 0.05 versus control; ^∗∗^*p* < 0.05 versus EtOH; ^∗∗∗^*p* < 0.05 versus EtOH + LPS.

Because ethanol oxidation further leads to the generation of ROS and oxidative stress, we considered it important to study the effect of T*β*4 on ethanol- and LPS-mediated oxidative stress by measuring ROS activity and lipid peroxidation, as well as the antioxidants, reduced GSH and manganese superoxide dismutase (Mn-SOD). Both EtOH alone or in combination with LPS significantly increased the levels of intracellular ROS by 48% (*p* < 0.05) and 54% (*p* < 0.05), respectively, which was markedly reduced by T*β*4 by 41% (*p* < 0.05) and 10% (*p* < 0.05) as compared to EtOH and EtOH + LPS groups, respectively (Figure 2(a)). Reduced GSH, an intracellular antioxidant, was decreased nominally by 20% in EtOH group and more significantly by 38% (*p* < 0.05) in EtOH + LPS group. T*β*4 administration restored the level of GSH with a 20% and 40% (*p* < 0.05) increase as compared to EtOH and EtOH + LPS groups, respectively (Figure 2(b)). Similarly, the protein expression of the antioxidant enzyme, Mn-SOD, was also similarly decreased in both EtOH- and EtOH + LPS-treated groups by about 38% (*p* < 0.05) that was restored over the control levels after T*β*4 treatment, with an approximately 50% (*p* < 0.05) increase as compared to EtOH and EtOH + LPS groups (Figure 2(c)). Furthermore, the protein expression of the lipid peroxidation product, 4-HNE, was markedly increased by EtOH alone or with LPS by 200% (*p* < 0.05) and 480% (*p* < 0.05), respectively, over the control levels. In contrast, T*β*4 prevented this increase by decreasing the protein expression of 4-HNE by 48% (*p* < 0.05) and 62% (*p* < 0.05) as compared to EtOH and EtOH + LPS groups, respectively (Figure 2(d)).

### 3.3. T*β*4 Prevents the EtOH- and LPS-Mediated Activation of NF*κ*B and the Induction of Proinflammatory Cytokines

**Figure 3:**
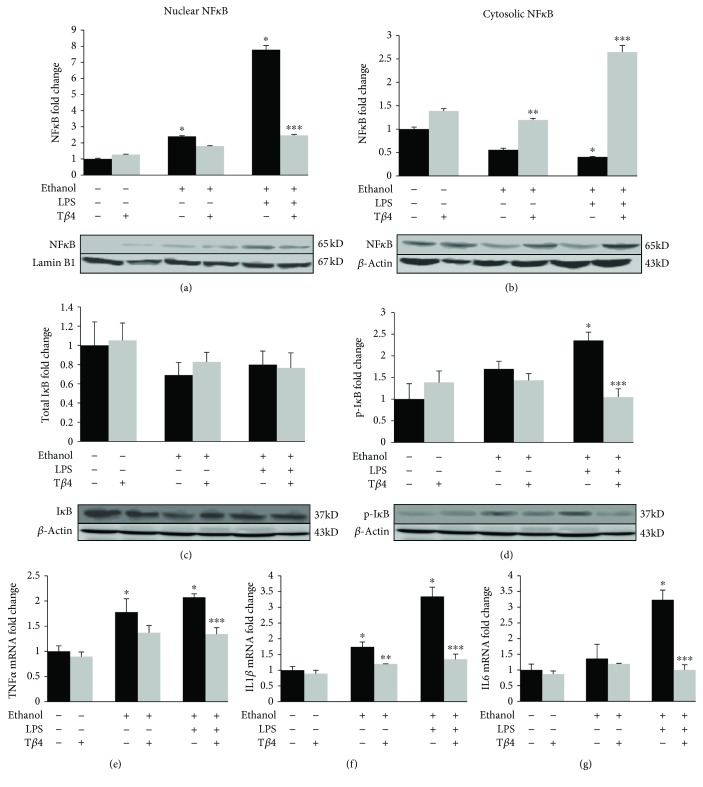
Effect of T*β*4 on EtOH- and LPS-induced activation of NF*κ*B and proinflammatory cytokine induction. Nuclear, cytosolic, or total protein was extracted from whole liver tissue from mice of various groups to determine the protein expression of (a) nuclear NF*κ*B, (b) cytosolic NF*κ*B, (c) total I*κ*B, and (d) p-I*κ*B by Western blot analysis. Total RNA was extracted from whole liver tissue from mice of various groups to determine mRNA expression using quantitative RT-PCR of (e) TNF*α*, (f) IL-1*β*, and (g) IL-6. All values are means of triplicate experiments ± SE and were corrected for loading differences after reprobing with lamin B1 (nuclear protein) or *β*-actin (cytosolic or total protein) or S18 (mRNA). ^∗^*p* < 0.05 versus control; ^∗∗^*p* < 0.05 versus EtOH; ^∗∗∗^*p* < 0.05 versus EtOH + LPS.

The activation of NF*κ*B and its translocation to the nucleus is an essential step for the activation of Kupffer cells and the production of proinflammatory cytokines [34, 35]. As illustrated in Figures 3(a) and 3(b), the administration of EtOH without or with LPS caused an increase in nuclear NF*κ*B by 140% (*p* < 0.05) and 677% (*p* < 0.05), respectively, over the control levels with a concomitant decrease in cytosolic NF*κ*B by about 45% (*p* < 0.05) and 60% (*p* < 0.05), respectively. T*β*4 significantly blocked these damaging effects by reversing the EtOH- and/or LPS-induced nuclear translocation of NF*κ*B by 25% and 70% (*p* < 0.05) and concomitantly increasing cytosolic NF*κ*B by 53% (*p* < 0.05) and 85% (*p* < 0.05) with respect to EtOH and EtOH + LPS groups, respectively (Figures 3(a) and 3(b)). These changes were associated with a change in the phosphorylation status of I*κ*B. As illustrated in Figures 3(c) and 3(d), while EtOH and LPS treatment showed no significant changes in the expression of total I*κ*B (Figure 3(c)), phosphorylation of I*κ*B was increased by 70% (*p* < 0.05) and 135% (*p* < 0.05) in EtOH and EtOH + LPS groups, respectively, over the control level, and T*β*4 administration resulted in its decrease by 15% and 56% (*p* < 0.05) as compared to EtOH and EtOH + LPS, respectively (Figure 3(d)). The activation of NF*κ*B further resulted in the induction of proinflammatory cytokine gene expression. EtOH administration significantly upregulated TNF-*α* (Figure 3(e)) and IL1*β* (Figure 3(f)) by 78% (*p* < 0.05) and 74% (*p* < 0.05), respectively, over the control, while the addition of LPS to EtOH upregulated TNF-*α* and IL1*β* by 107% (*p* < 0.05) and 234% (*p* < 0.05), respectively (Figures 3(e) and 3(f)). Although EtOH alone did not significantly upregulate the mRNA levels of IL6, administration of both EtOH and LPS caused a 223% (*p* < 0.05) increase in IL6 mRNA (Figure 3(g)). These EtOH- and LPS-mediated changes were blunted by T*β*4. TNF*α*, IL1*β*, and IL6 mRNA were downregulated by T*β*4 by 35% (*p* < 0.05), 60% (*p* < 0.05), and 69% (*p* < 0.05) as compared to EtOH + LPS group, respectively (Figures 3(e) and 3(g)).

### 3.4. T*β*4 Prevents the EtOH- and LPS-Induced Activation of Hepatic Stellate Cells and Fibrogenesis

**Figure 4:**
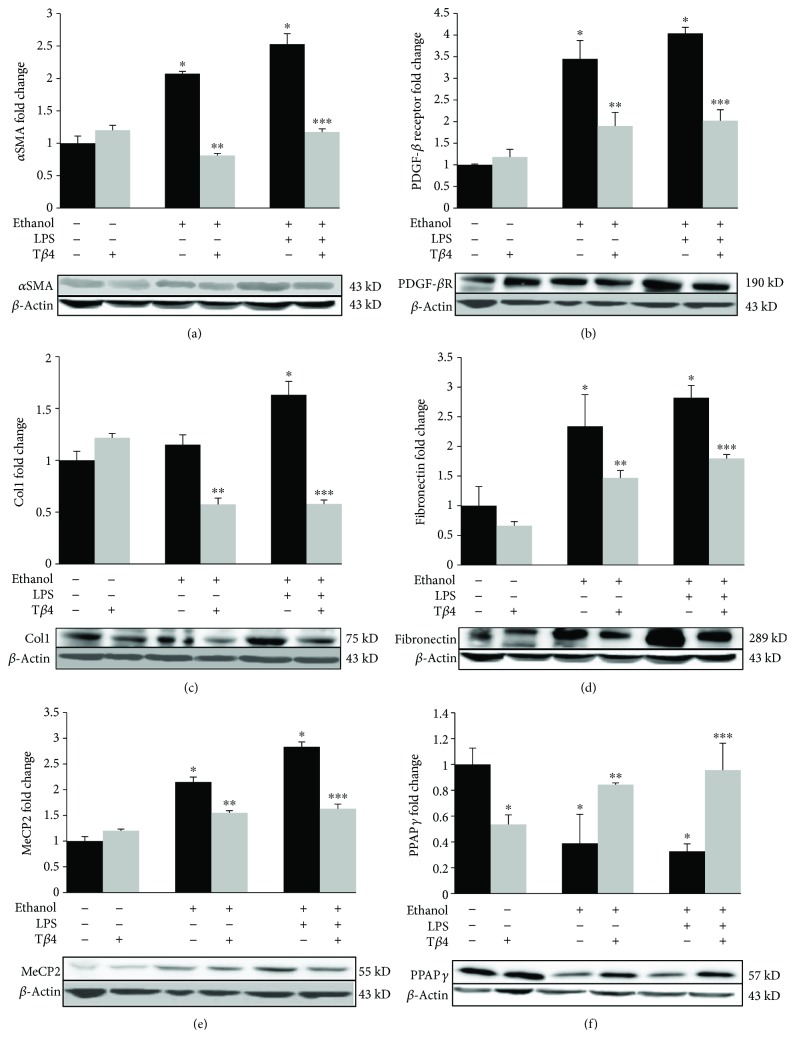
Effect of T*β*4 on EtOH- and LPS-induced fibrogenesis. Total protein was extracted from whole liver tissue from mice of various groups to determine the protein expression of (a) *α*SMA, (b) PDGF-*β* receptor, (c) collagen 1, (d) fibronectin, (e) MeCP2, and (f) PPAR*γ* by Western blot analysis. All values are means of triplicate experiments ± SE and were corrected for loading differences after reprobing with *β*-actin. ^∗^*p* < 0.05 versus control; ^∗∗^*p* < 0.05 versus EtOH; ^∗∗∗^*p* < 0.05 versus EtOH + LPS.

The cytokines and chemokines derived from Kupffer cells, such as TGF-*β*1, PDGF, TNF-*α*, and IL-1*β* as well as acetaldehyde-mediated oxidative stress, act as a stimulus to induce the activation of HSC and the subsequent fibrogenesis [34, 36]. The expressions of *α*SMA and PDGF-*β* receptor are the hallmarks of HSC activation and their transformation into the myofibroblasts [22, 37]. Therefore, to investigate the effect of T*β*4 on EtOH- and LPS-mediated fibrogenesis, we determined the protein expression of *α*SMA and PDGF-*β* receptor. EtOH without or with LPS significantly increased the expression of the *α*SMA by 107% (*p* < 0.05) and 153% (*p* < 0.05) over the control that was decreased by 61% (*p* < 0.05) and 54% (*p* < 0.05), respectively, after T*β*4 treatment (Figure 4(a)). Likewise, PDGF-*β* receptor expression was also markedly increased by 245% (*p* < 0.05) in EtOH group and by 304% (*p* < 0.05) in EtOH + LPS group that was blocked by T*β*4 by 45% (*p* < 0.05) and 50% (*p* < 0.05), respectively (Figure 4(b)). Moreover, as shown in Figures 4(c) and 4(d), EtOH increased the protein expression of ECM markers, collagen 1 by 15% and fibronectin by 134% (*p* < 0.05). Addition of LPS to EtOH led to a further increase in collagen 1 expression by 63% (*p* < 0.05) and fibronectin by 182% (*p* < 0.05). These changes were reversed by T*β*4 resulting in a 50% (*p* < 0.05) and 65% (*p* < 0.05) decrease in collagen 1 expression (Figure 4(c)) and a 37% (*p* < 0.05) decrease in fibronectin expression (Figure 4(d)) as compared to EtOH- and EtOH + LPS-treated groups, respectively. Furthermore, EtOH and EtOH + LPS markedly increased the expression of epigenetic repressor of adipogenic phenotype, MeCP2, by 115% (*p* < 0.05) and 183% (*p* < 0.05), respectively (Figure 4(e)), which in turn, coordinately decreased the expression of the adipogenic regulator, PPAR*γ*, by about 61% and 67% (*p* < 0.05), respectively (Figure 4(f)). Treatment with T*β*4 resulted in a significant decrease in MeCP2 expression by 28% (*p* < 0.05) and 42% (*p* < 0.05) as compared to EtOH and EtOH + LPS groups, respectively (Figure 4(e)), whereas the expression of PPAR*γ* was restored close to the control by T*β*4 treatment (Figure 4(f)).

### 3.5. T*β*4 Prevents the EtOH- and LPS-Induced Hepatic Fibrosis

**Figure 5:**
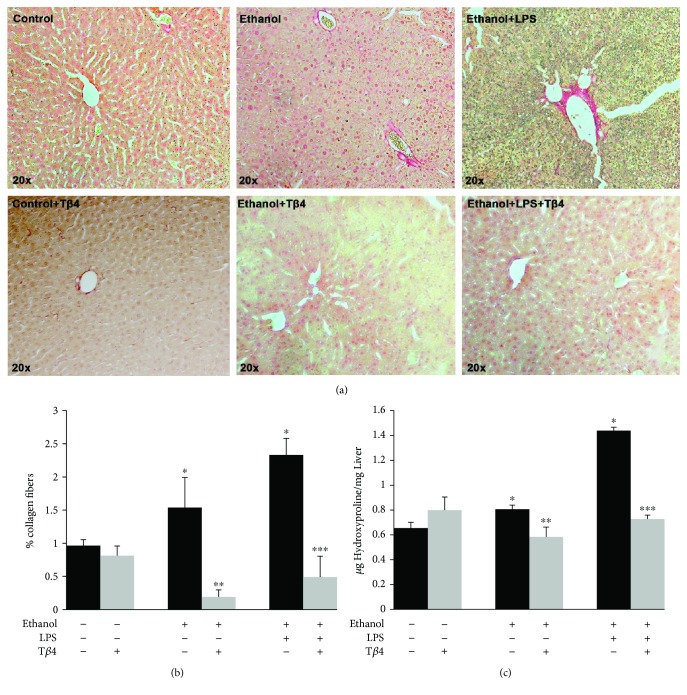
Effect of T*β*4 on EtOH- and LPS-induced fibrosis. Liver tissue from various groups was used to determine (a) Sirius Red staining, magnification, 20x, (b) quantification of percentage of collagen fibers, and (c) hydroxyproline content (indicative of collagen fibers) by biochemical assay to determine the extent of fibrosis. All values are means of triplicate experiments ± SE. ^∗^*p* < 0.05 versus control; ^∗∗^*p* < 0.05 versus EtOH; ^∗∗∗^*p* < 0.05 versus EtOH + LPS.

To further investigate if the changes in the activation of HSC result in fibrosis, we examined the extent of fibrosis in the various groups by performing Sirius Red staining that stains for collagen fibers. As illustrated in Figures 5(a) and 5(b), EtOH alone or in combination with LPS caused a 60% (*p* < 0.05) and 140% (*p* < 0.05) increase in Sirius Red staining, respectively, which was blocked by T*β*4 treatment by 88% (*p* < 0.05) and 80% (*p* < 0.05) as compared to EtOH and EtOH + LPS groups, respectively. These results were confirmed by determining the hydroxyproline content, which is also an indication for the collagen content [38].  Figure 5(c) shows that EtOH without or with LPS increased the hydroxyproline content by 25% (*p* < 0.05) and 120% (*p* < 0.05), respectively. T*β*4 treatment decreased the EtOH- and EtOH + LPS-mediated effect by 28% and 50% (*p* < 0.05), respectively, thus reducing the extent of fibrosis.

## 4. Discussion

Great progress has been made in understanding the molecular mechanisms involved in the development of ALD [2, 39, 40], yet therapeutic treatments still remain limited. There are several therapies for treating chronic liver damage such as antivirals for hepatitis B or C viral infection, HGF for hepatocyte regeneration, and corticosteroids for steatohepatitis [41, 42]; however, treatment for ALD is severely lacking, because most existing therapies only focus on the prevention and treatment of consequences and complications that arise during ALD [34, 43]. Moreover, 40% of the patients develop end-stage liver disease with significant hepatic fibrosis and cirrhosis, in which case, liver transplantation is the only curative approach [34]. A substantial number of lives depend on receiving liver tissue for transplantation; however, due to the severe shortage in donor availability, each year, over 1000 patients die waiting for a liver to become available. Hence, there is an urgent need for therapies for not only prevention and early intervention to arrest the disease progression but also to effectively regenerate the remaining healthy liver so that the patient can be reasonably functional before they can fully recover with a liver transplantation [43].

It is well known that obesity and alcohol consumption are known to synergistically aggravate the progression of liver disease [44]. Numerous studies have established that a high *ω*-3 polyunsaturated fatty acid (PUFA) diet is more harmful in causing alcoholic liver injury than saturated fat diets [45–49]. We have previously shown that low *ω*-3 PUFA, but not high *ω*-3 PUFA, attenuates alcoholic liver injury [50]. Moreover, Chang et al. have demonstrated that while there was no significant increase in serum AST and ALT levels in chow-fed mice that were administered with a single dose of ethanol (5 g/kg) gavage, there was a significant increase in the serum AST and ALT levels in high-fat diet fed mice that were administered with a single dose ethanol (5 g/kg) gavage [51].

Emerging evidence suggests that alcohol per se is not solely responsible for the initiation and/or progression of ALD, and endotoxin (LPS) has become a prime suspect as a key contributor to the onset of advanced liver disease accompanying chronic alcoholism [3–6]. In this study, we used two *in vivo* liver injury models, EtOH-mediated model was adapted from the NIAAA model by Gao et al. [29] that includes 4-week chronic EtOH feeding and an acute dose of EtOH (5 g/kg body wt, gavage) and a hybrid model that combines LPS with EtOH, adapted from the 2-hit EtOH/LPS model by Hoek et al. [4]. The hybrid model of EtOH and LPS mimics the human disease by combining EtOH-mediated oxidative stress caused by acetaldehyde, the toxic product of EtOH, and leaky gut leading to the release of endotoxin (LPS), which induces proinflammatory cytokines resulting in inflammation and fibrosis [2, 4]. This model significantly increased liver injury and inflammation and produced moderate to severe fibrosis as compared to other EtOH-mediated liver injury models [29, 52], which showed negligible or mild fibrosis. Interestingly, EtOH and LPS have distinct effects on some aspects of liver injury while an additive effect on others. EtOH alone mediates the increase in ROS, a known contributor of ROS from the production of acetaldehyde [53], and LPS has little or no role in further increasing ROS levels. On the other hand, in case of the nuclear translocation of NF*κ*B and the induction of proinflammatory cytokines, EtOH only shows a modest increase, whereas LPS causes a more significant increase in the expression of these parameters. This is also in accordance with the well-established fact that LPS is a major contributor for inflammation in ALD [54, 55]. In contrast, our results show that EtOH and LPS cause an additive effect on the expression of fibrogenic markers.

T*β*4, the main G-actin-sequestering protein, is the most abundant and highly conserved member of the thymosin family of proteins. Due to the lack of a stable folded structure in aqueous solution, T*β*4 functions as an intrinsically unstructured protein. It binds to several partner proteins to acquire a stable folded structure, thus offering many possibilities for interaction with multiple partners [11]. Therefore, T*β*4 is implicated in a diverse range of physiological effects such as wound healing, tissue repair, and angiogenesis [11]. It also plays a protective role against inflammation and fibrosis after myocardial infarction, corneal injury, and kidney damage [56]. Although T*β*4 is expressed in the liver, the specific cell types that express T*β*4 are not well established. Whereas one study showed that T*β*4 is expressed in hepatocytes from healthy human liver [57], another study reported that T*β*4 is expressed in Kupffer cells in the damaged liver [58]. Furthermore, Kim et al. have demonstrated that T*β*4 is expressed by HSC in chronically damaged liver [59].

Although hepatocytes have efficient mechanisms to regulate intracellular levels of ROS and antioxidant system, their ability to maintain redox balance can fail in case of chronic insult and extensive damage caused by EtOH and LPS [2, 60]. Wei et al. [17] have demonstrated that T*β*4 prevents oxidative stress by targeting the antioxidant system and thereby preventing the loss of mitochondrial membrane potential in cardiac fibroblasts. In corneal epithelial cells, it reduces EtOH-induced proapoptotic effects by decreasing cytochrome c release from the mitochondria and caspase activation [14]. In the present study, T*β*4 prevented EtOH- and LPS-induced increase in ROS by altering the expression of Mn-SOD and reduced GSH, thereby preventing oxidative stress.

The liver is the central organ for the production of cytokines that are released by Kupffer cells, the resident hepatic macrophages, to induce an inflammatory response after injury. In particular, LPS induces the activation of Kupffer cells via Toll-like receptor 4 binding, leading to the generation of proinflammatory cytokines [2, 4]. Pretreatment with T*β*4 causes a decrease in nuclear NF*κ*B protein levels, NF*κ*B activity, p65 subunit phosphorylation, and nuclear translocation in corneal epithelial cells stimulated with TNF-*α* [19]. In this study, T*β*4 inhibited the activation of Kupffer cells by preventing the translocation of NF*κ*B from the cytoplasm to the nucleus, where it initiates the transcription of proinflammatory cytokines. The process of NF*κ*B translocation to the nucleus is regulated by the phosphorylation of I*κ*B, which was blunted by T*β*4. Furthermore, the anti-inflammatory property of T*β*4 was confirmed by its prevention of EtOH- and LPS-mediated induction of proinflammatory cytokines. To the best of our knowledge, the present study is the first one to demonstrate this anti-inflammatory property of T*β*4, although others have reported its beneficial effect in protecting against cardiac, renal, and corneal injury [13, 15–18, 20, 56].

Injury, inflammation, and oxidative stress often lead to fibrosis via the transdifferentiation of HSC into myofibroblasts ([37]). Several studies have explored the role of T*β*4 in the activation of HSC. Hong et al. demonstrated that T*β*4 inhibited HSC activation by suppressing Notch signaling, thus attenuating liver fibrosis [61]. Others have shown that T*β*4 regulates HSC activation via hedgehog signaling [62]. Additionally, depletion of T*β*4 has shown to promote the proliferation, migration, and activation of HSC [63]. This is in accordance with our previous work demonstrating that T*β*4 prevented activation, proliferation, and migration of HSC via inhibiting the phosphorylation of Akt signaling [22]. Moreover, Barnaeva et al. showed that T*β*4 treatment upregulated the gene expression of HGF and downregulated the expression of PDGF-*β* receptor in cultured HSC [64]. In this study, T*β*4 diminished EtOH- and LPS-mediated transdifferentiation of quiescent HSC to myofibroblasts and the subsequent fibrogenesis by preventing the increase in HSC activation pathway genes and ECM proteins. We have further shown that the mechanism of action of T*β*4 in attenuating fibrogenesis is mediated by the suppression of MeCP2 and an increase of PPAR*γ* expression, thereby maintaining the HSC in their quiescent state. Surprisingly, there is a decrease in PPAR*γ* expression in the T*β*4-treated control. However, this result supports other reports which show that without liver injury, silencing T*β*4 results in an increase in PPAR*γ* expression, and our results show that without liver injury, exogenously administering T*β*4 results in a decrease in PPAR*γ* expression [59, 62]. T*β*4 also reduced the extent of fibrosis as measured by the percentage of collagen fibers and the amount of hydroxyproline in the liver tissue. These results are consistent with our previous finding that T*β*4 protects against carbon tetrachloride-induced acute liver injury in rats [23]. Nevertheless, in-depth molecular mechanism by which T*β*4 exerts its protective effects remains to be explored. One explanation could be the interaction of T*β*4 with PDGF-BB. It is known that activated Kupffer cells secrete a variety of cytokines and growth factors such as PDGF-BB and TGF-*β*1 [34]. These growth factors, along with the acetaldehyde-induced oxidative stress, stimulate HSC activation and the fibrogenic cascade. Indeed, our previously published work showed that T*β*4 prevented PDGF-BB-induced proliferation and fibrogenesis of cultured HSC by inhibiting the phosphorylation of Akt [22]. However, mechanistic studies need to be carried out to establish the underlying molecular mechanism by which T*β*4 exerts antioxidant, anti-inflammatory, and antifibrotic actions during alcoholic liver injury.

## 5. Conclusion

In conclusion, the present study is the first to demonstrate the antioxidant, anti-inflammatory, antifibrotic, and regenerative potential of T*β*4 during chronic EtOH- and LPS-induced liver injury. T*β*4 presumably accomplishes all of these actions by inhibiting the activation of NF*κ*B signaling and simultaneously preventing the activation of HSC and the fibrogenic cascade by suppressing MeCP2, *α*SMA, and PDGF-*β* receptor and upregulating PPAR*γ*, thereby preventing the transdifferentiation of the myofibroblasts and thus preventing fibrosis.

## Data Availability

The data used to support the findings of this study are available from the corresponding author upon request.

## Abbreviations

ALD: Alcoholic liver disease
LPS: Lipopolysaccharide
EtOH: Ethanol
NF*κ*B: Nuclear factor kappa B
TNF*α*: Tumor necrosis factor alpha
IL1*β*: Interleukin 1 beta
IL6: Interleukin 6
PDGF-BB: Platelet-derived growth factor BB
ROS: Reactive oxygen species
4-HNE: 4-Hydroxy-2-nonenal
HSC: Hepatic stellate cells
ECM: Extracellular matrix
PPAR*γ*: Peroxisome proliferator-activated receptor gamma
MeCP2: Methyl-CpG-binding protein 2
*α*SMA: Alpha smooth muscle actin
T*β*4: Thymosin beta 4.
